# Supplementary material for: Chemotherapy-induced complement signaling modulates immunosuppression and metastatic relapse in breast cancer
Source: Nat Commun. 2022 Oct 2;13:5797. doi: 10.1038/s41467-022-33598-x (PMC9527249; doi:10.1038/s41467-022-33598-x)
Supplement: Supplementary file 1 — Supplementary Information [file 41467_2022_33598_MOESM1_ESM.pdf]

Supplementary Information for:

Chemotherapy-induced complement signaling modulates immunosuppression and metastatic relapse in breast cancer

Lea Monteran*[1], Nour Ershaid*[1], Hila Doron[1], Yael Zait[1], Ye'ela Scharff[1], Shahar Ben-Yosef[1], Camila Avivi[2], Iris Barshack[2], Amir Sonnenblick[3] and Neta Erez[1#].

* These authors contributed equally to this manuscript.

# Corresponding author: netaerez@tauex.tau.ac.il

The PDF include:

Supplementary Figures:

Supplementary Fig. 1. Cytotoxic treatment reduces fibrogenic properties of lung fibroblasts while disrupting immune milieu in lungs.

Supplementary Fig. 2. Gating strategy used for analysis of immune-cell infiltration and exhaustion markers.

Supplementary Fig. 3. Adjuvant chemotherapy for EO771-based breast cancer model reshapes the lung immune landscape.

Supplementary Fig. 4. Doxorubicin affect both immune milieu and ECM deposition at early metastatic stage.

Supplementary Fig. 5. Adjuvant AC regimen (doxorubicin and cyclophosphamide) is ineffective in restricting lung metastasis of breast cancer.

Supplementary Fig. 6. Adjuvant chemotherapy induced transcriptional reprogramming in lung fibroblasts.

Supplementary Fig. 7. Lung CAFs are a major source of complement components in murine and human carcinomas.

Supplementary Fig. 8. Complement-signaling activation occurs following doxorubicin treatment but not cisplatin.

Supplementary Fig. 9. Complement blockade alone is inefficient in curbing pulmonary metastasis of breast cancer.

Supplementary Table:

Supplementary Table. 1. Primer's list.

Supplementary Figures:

Monteran_Ershaid_ Supplementary Fig. 1

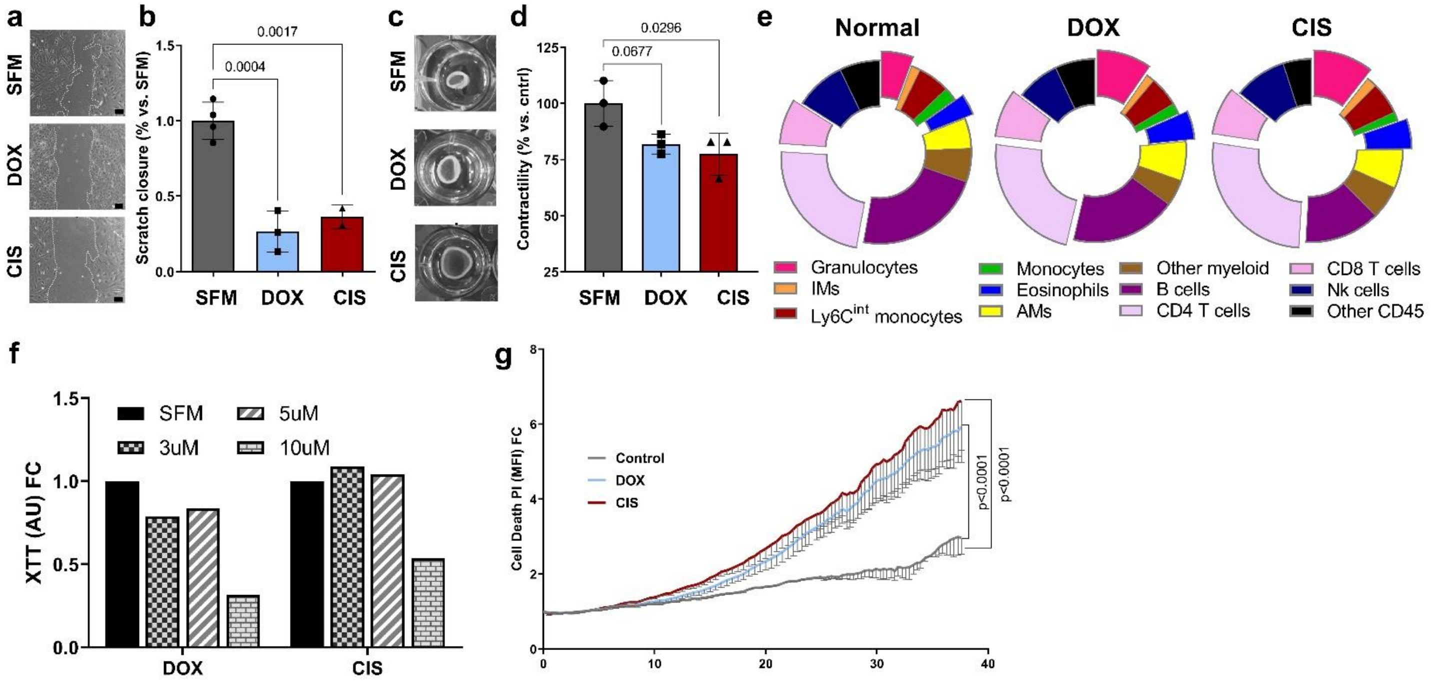

Supplementary Fig. 1. Cytotoxic treatments reduce fibrogenic properties of lung fibroblasts while disrupting immune milieu at lungs. (a, b) Scratch closure assay. NLFs were treated for 24h with 5uM Doxorubicin, 1uM Cisplatin, or control medium. **(a)** Representative images of scratch closure assay. Scale bars, 100µm **(b)** Quantification of scratch area. n=3 independent experiments. **(c, d)** Collagen contraction assay. NLFs were for 24h with 5µM Doxorubicin, 1µM Cisplatin, or control medium. **(c)** Representative images of collagen contraction assay. **(d)** Quantification of collagen contraction. n=3 independent experiments. In **(b, d)** data are presented as mean ± s.d of technical repeats, normalized to control; P-values were calculated using One-way ANOVA test. *$p < 0.05$. **(e)** Immune landscape of lungs following single dose chemotherapy (doxorubicin 5mg/kg or cisplatin 5mg/kg) or PBS was analyzed using flow cytometry. Donut plots depicting proportions of various immune cell populations in lungs. n=5 mice per group. **(f, g)** Evaluation of chemotherapy induced-cytotoxicity. 4T1 cells were incubated with doxorubicin or cisplatin at specified concentrations (3µM, 5µM, or 10µM) or control media (SFM) for 48h. Cytotoxicity was evaluated using **(f)** XTT-based assay, n=2 independent experiments, and **(g)** Cytotoxicity was evaluated using PI staining measured by the Incucyte system. n=3 technical repeats, P-values were calculated using One-way ANOVA test. Source data are provided as a Source Data file.

Monteran_Ershaid_Supplementary Fig. 2

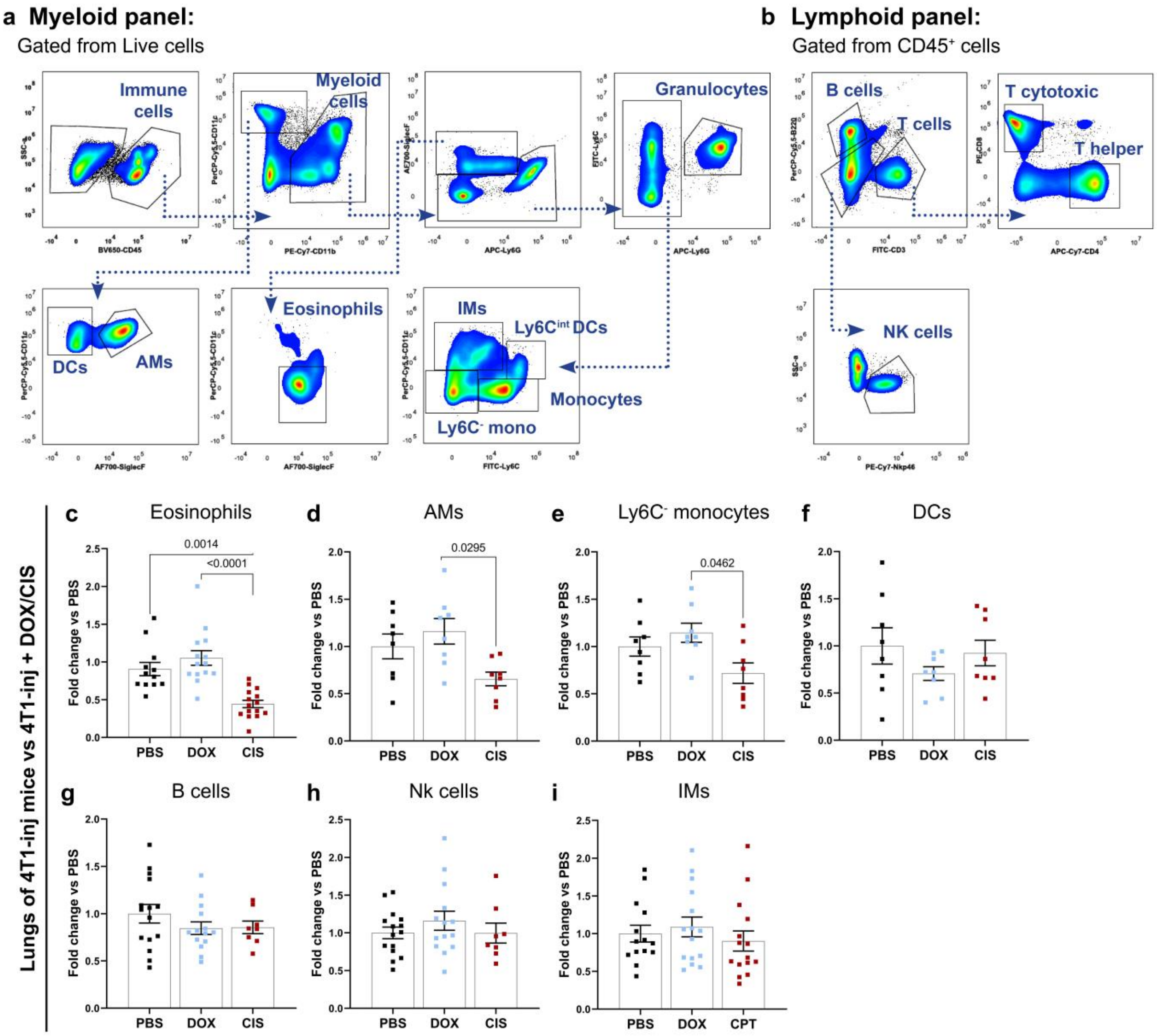

Supplementary Fig. 2. Analysis of immune cell infiltration and exhaustion markers. (a, b) Gating strategy used for proportion analysis of myeloid (a) and lymphoid (b) immune cell populations. **(c-i)** Flow cytometry analysis of the immune landscape in lungs at early metastatic stage in the 4T1 model, 24h following treatment with the last dose of doxorubicin (5mg/kg), cisplatin (5mg/kg), or PBS. **(c)** n=12, 14 and 15. **(d-f)** n=8 for each groups **(g)** n=15, 14 and 8. **(h)** n=15, 14 and 8. **(i)** n=14, 15 and 15 mice in control, doxorubicin, and cisplatin, respectively. Data presented as absolute number of cells per lungs. Error bars represent s.e.m; P-values were calculated using Kruskal-Wallis test for multiple comparisons. Source data are provided as a Source Data file.

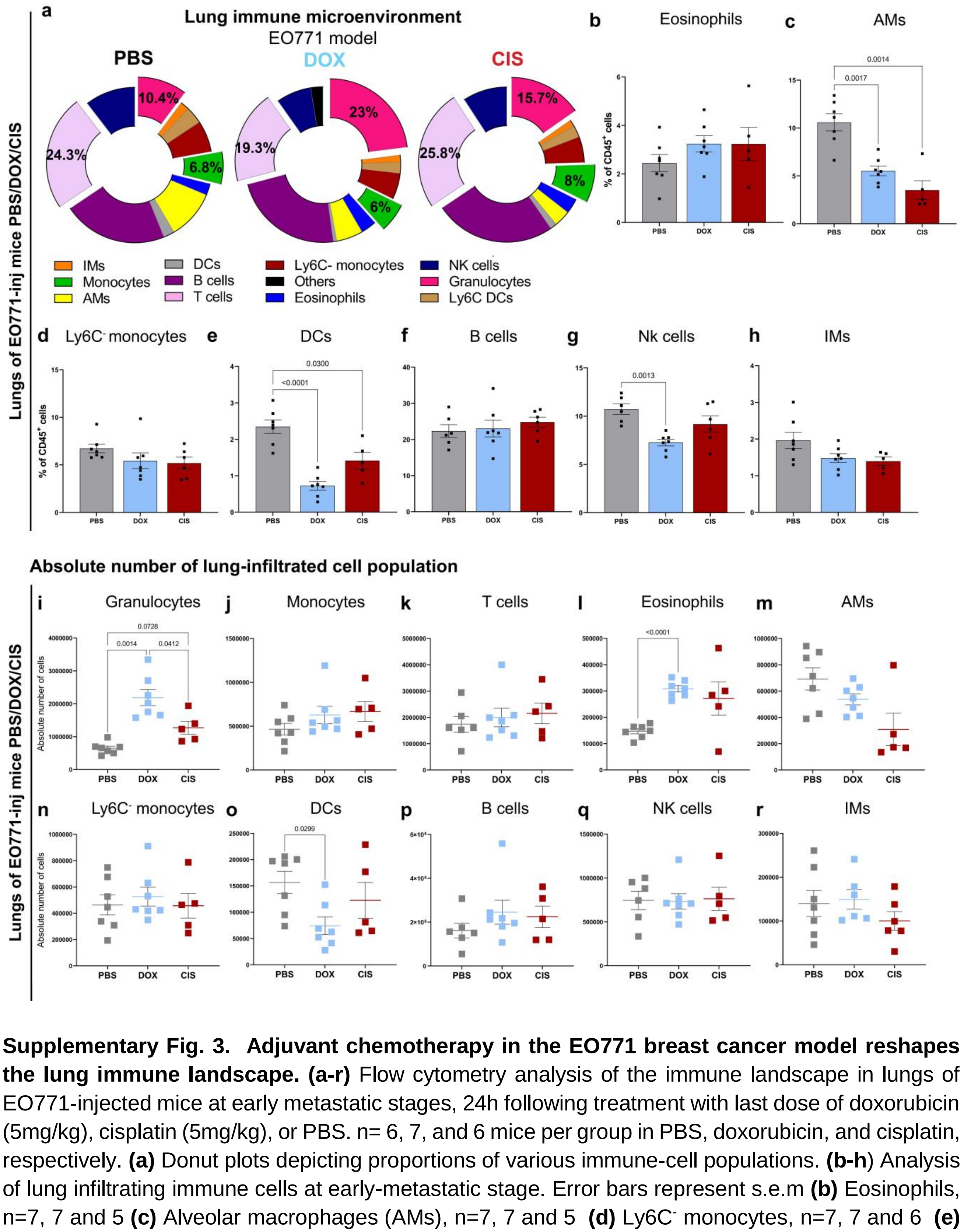

Supplementary Fig. 3. Adjuvant chemotherapy in the EO771 breast cancer model reshapes the lung immune landscape. (a-r) Flow cytometry analysis of the immune landscape in lungs of EO771-injected mice at early metastatic stages, 24h following treatment with last dose of doxorubicin (5mg/kg), cisplatin (5mg/kg), or PBS. n= 6, 7, and 6 mice per group in PBS, doxorubicin, and cisplatin, respectively. **(a)** Donut plots depicting proportions of various immune-cell populations. **(b-h)** Analysis of lung infiltrating immune cells at early-metastatic stage. Error bars represent s.e.m **(b)** Eosinophils, n=7, 7 and 5 **(c)** Alveolar macrophages (AMs), n=7, 7 and 5 **(d)** Ly6C$^-$ monocytes, n=7, 7 and 6 **(e)** Dendritic cells (DCs), n=7, 7 and 5 **(f)** B cells, n=6, 7 and 5 **(g)** NK cells, n=6, 7 and 5 **(h)** and interstitial macrophages (IMs), n=7, 7 and 5 for PBS, DOX or CIS. Data presented are percentage of

CD45$^+$ cells, normalized to PBS. P-values were calculated using Kruskal-Wallis test for multiple comparisons. (**i-r**) Absolute numbers of specified lung-infiltrating immune cells. **(i, j)** n=7, 7 and 5; **(k)** n=6, 7 and 5; **(l-o)** n=7, 7 and 5; **(p,q)** n=6, 7 and 5; **(r)** n=7, 6 and 6 for PBS, DOX and CIS. Error bars represent s.e.m. P-values were calculated using Kruskal-Wallis test for multiple comparisons. Source data are provided as a Source Data file.

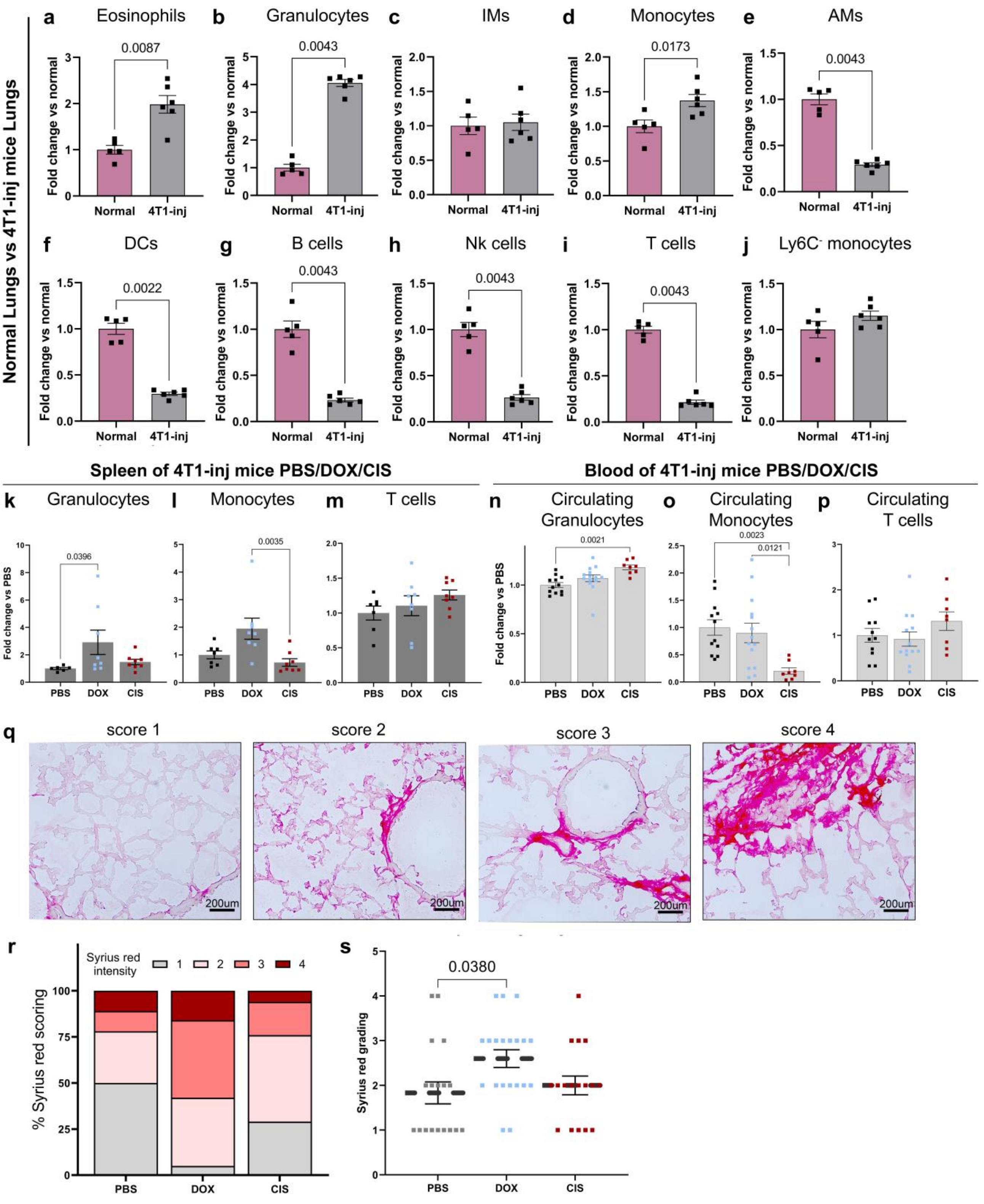

Supplementary Fig. 4. Doxorubicin affects both immune milieu and ECM deposition at early metastatic stage.

(a-j) Flow cytometry analysis of the immune landscape in lungs of 4T1-injected mice at early metastatic stages as compared with non-tumor bearing normal mice. n=5 and 6 for normal and 4T1-

injected groups, respectively. Data presented as mean percentage out of $CD45^+$ cells, normalized to normal control group. Error bars represent s.e.m; P-values were calculated using Mann-Whitney test. **(k-m)** Flow cytometry analysis of proportion of **(k)** Granulocytes, **(i)** Monocytes, and **(m)** T cells in spleens of 4T1-injected mice following treatment with DOX (n= 8), CIS (n= 8), or PBS as control (n= 6). **(n-p)** Flow cytometry analysis of proportion of blood circulation **(n)** Granulocytes, **(o)** Monocytes, and **(p)** T cells in 4T1-injected mice following treatment with DOX (n= 13), CIS (n= 8), or PBS as control (n= 11). For (k-p), data are presented as mean percentage out of $CD45^+$ cells, normalized to PBS group of each organ. Error bars represent s.e.m; P-values were calculated using Kruskal-Wallis test for multiple comparisons. **(q-s)** Sirius Red staining of lungs from 4T1-injected mice at early metastatic stages, 24h following DOX, CIS, or PBS treatments, n= 3 mice per group. **(q)** Representative images of different staining intensity of Sirius Red. Staining intensity scale 1-4, 4=strongest staining. **(r, s)** Quantification of collagen staining in lungs presented as % of fields of view analyzed (r) or average grade of intensity (s). Each dot represents a field of view, at least 3 fields of view per mouse were analyzed. Error bars represent s.e.m. n=18, 20 and 17 for PBS, DOX and CIS, respectively. P-values were calculated using Kruskal-Wallis test for multiple comparisons. Source data are provided as a Source Data file.

Monteran_Ershaid_Supplementary Fig. 5

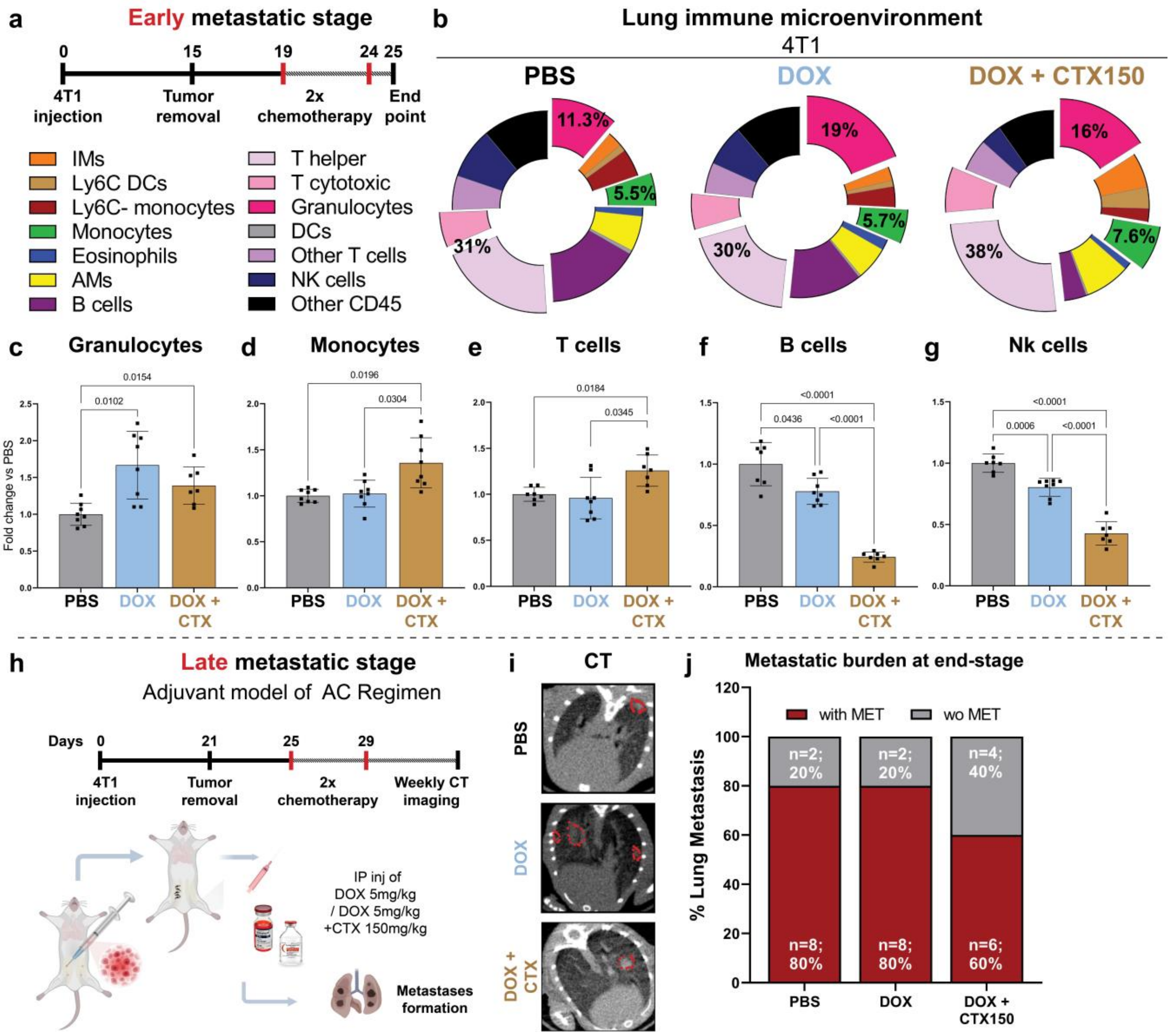

Supplementary Fig. 5. Adjuvant AC regimen (doxorubicin and cyclophosphamide) is ineffective in restricting lung metastasis of breast cancer. (a) Schematic illustration of TNBC mouse model combined with adjuvant AC chemotherapy. 4T1 cells were orthotopically injected into the mammary fat pad of BALB/c mice. For the early metastatic stage (a-g), tumors were resected 2w post-inoculation and mice received two cycles of adjuvant AC (5 mg/kg doxorubicin + 150mg/kg cyclophosphamide) or PBS as control. **(a-g)** Flow cytometry analysis of the immune landscape of lungs at early metastatic stages. **(b)** Proportions of various immune cell populations in lungs. Representative plots and quantification of altered populations: **(c)** Granulocytes, n=8, 8, and 7 **(d)** Monocytes, n=8 for each group **(e)** T cells, n=7, 8 and 7 **(f)** B cells, n=7, 8 and 7 and **(g)** NK cells, n=7, 8 and 7mice in control, doxorubicin, and AC-regimen groups, respectively. In (c-g), data are presented as mean percentage out of CD45^{+} cells, normalized to PBS group. Error bars represent s.e.m; P-values were calculated using Kruskal-Wallis test for multiple comparisons. **(h)** Adjuvant model of AC regimen in late stage metastasis used in (i-j). Following tumor removal (3w post-inoculation), mice received two cycles of chemotherapy, or PBS as control. Spontaneous lung metastases formation was monitored intravitally by CT imaging. **(i)** Representative CT scans depicting

lungs at late metastatic stages. Metastases are circled in red. **(j)** Metastatic incidence at end-stage in the 4T1 model, n= 10 mice per group. P-values were calculated using two tailed Chi square test. Graphical summary was designed using BioRender. Source data are provided as a Source Data file.

Monteran_Ershaid_Supplementary Fig. 6

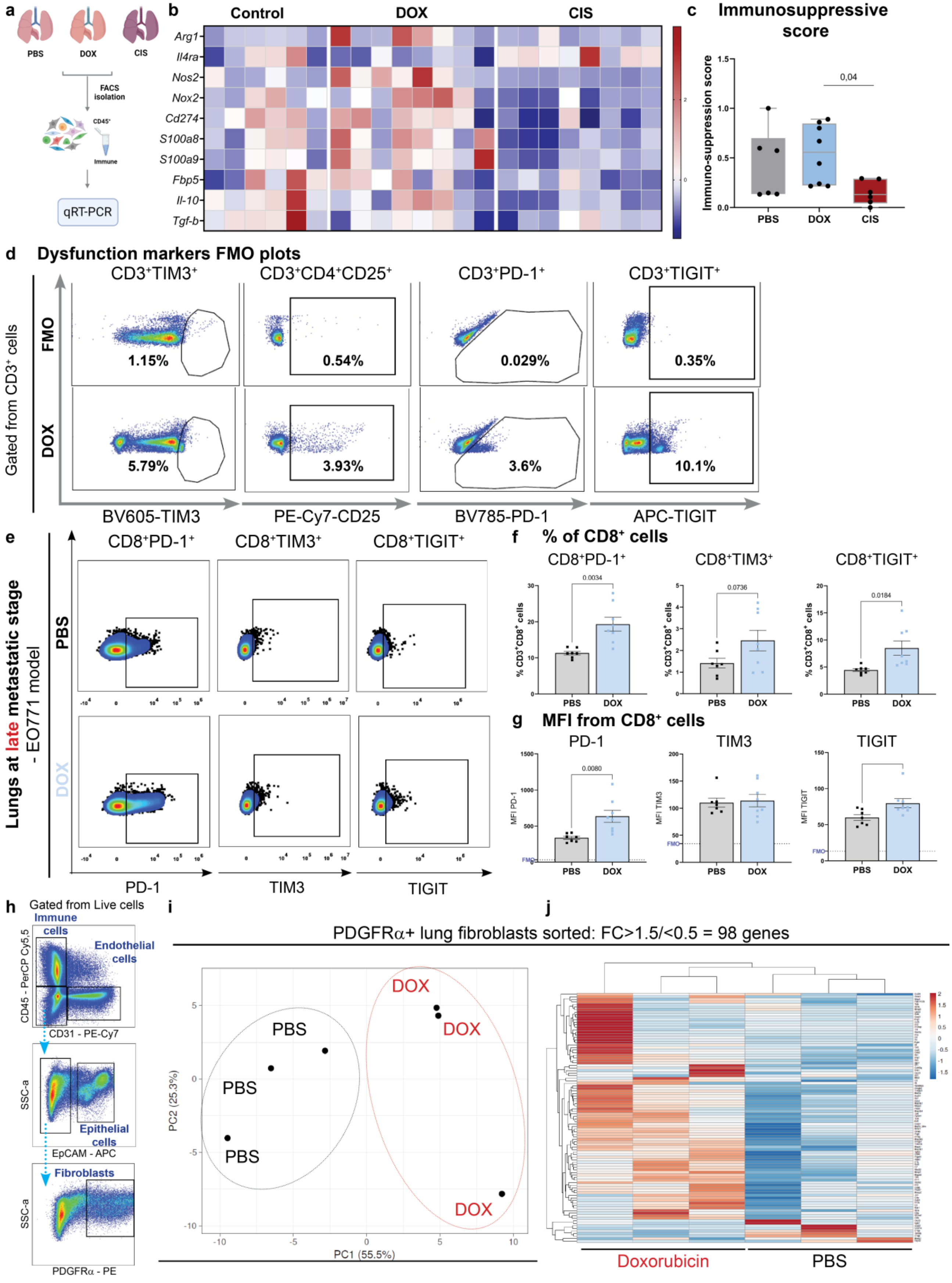

Supplementary Fig. 6. Adjuvant chemotherapy induces immunosuppression and transcriptional reprogramming in lung fibroblasts. (a) Schematic illustration of experimental procedure analyzed in (b-c). CD45^{+} immune cells were FACS sorted from early stage metastatic lungs of either PBS, DOX or CIS treated mice. Gene expression in sorted cells was analyzed by qRT-PCR. **(b)** Heatmap depicting gene expression of immunosuppression-related genes. n=7, 8 and 8 mice in control (PBS), doxorubicin (DOX), and cisplatin (CIS), respectively. **(c)** Expression of immunosuppression-related genes was combined into an “immunosuppressive score”. n=6, 8 and 6 mice in PBS, DOX and CIS respectively. Bars represent range, line represents mean. P-values were calculated using Two-way ANOVA test. **(d)** Gating strategy and representative FMO controls (Fluorescent Minus One) of immune dysfunction markers in CD8^{+} cytotoxic T cells. **(e)** Representative flow cytometry plots of CD3^{+}CD8^{+} T cells expressing dysfunction markers and quantification of changes. **(f)** Quantification of % CD8^{+} expressing dysfunction markers, data are presented as mean ± s.e.m. **(g)** PD-1, TIM3 and TIGIT protein expression presented as MFI, data are presented as mean ± s.e.m. (e-g) n=7, 8 mice in PBS and doxorubicin, respectively. P-values were calculated using two tailed Welch’s t-tests. **(h)** Gating strategy for FACS sorting of fibroblasts (PDGFR□$^{+}$), epithelial cells (EpCAM^{+}), endothelial cells (CD31^{+}) and immune cells (CD45^{+}) from lungs. Principal component analysis (PCA) **(i)** and hierarchical clustering **(j)** of 98 genes upregulated or downregulated in lung fibroblasts of doxorubicin-treated mice (dox) vs. control mice (pbs) based on fold change (FC) < |0.5| or (FC) > |1.5| identified in NanoString analysis. PCA plots and clustered heatmaps were prepared using ClustVis tool (https://doi.org/10.1093/nar/gkv468). (i, j) n=3 per group. Graphical summary was designed using BioRender. Source data are provided as a Source Data file.

Monteran_Ershaid_Supplementary Fig.7

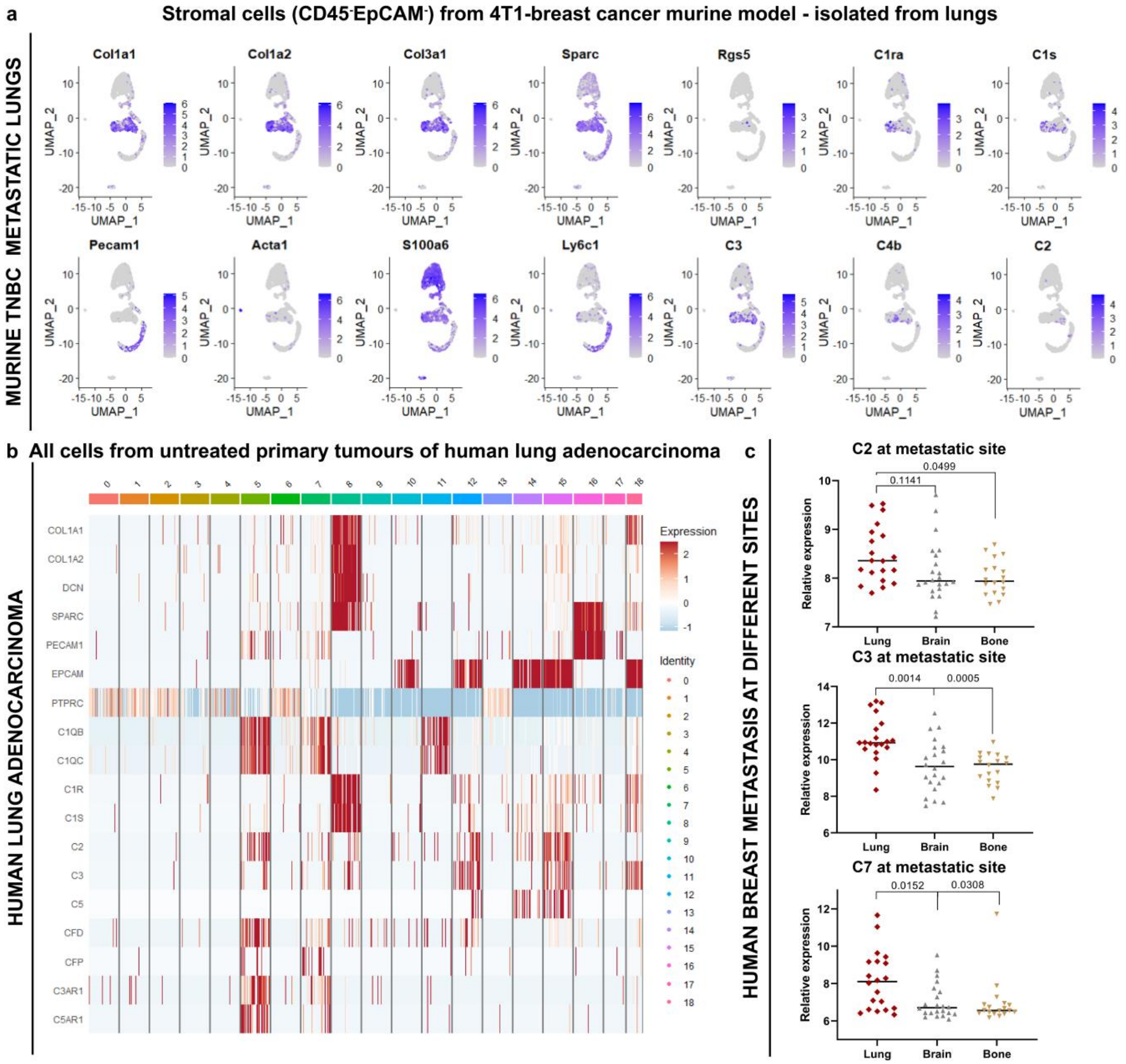

Supplementary Fig. 7. Lung CAFs are a major source of complement components in murine and human carcinomas. (a) Feature plots of expression distribution for selected cluster-specific genes and complement-related genes. Expression levels for each cell are color-coded and overlaid onto the UMAP plot. Data from GSE149636, a single-cell RNAseq database of the stromal compartment ($CD45^-EpCAM^-$ cells) in lungs of mice with 4T1 breast cancer. **(b)** Heatmap depicting expression of selected cluster-specific genes and complement-related genes derived from GSE123902, single-cell RNAseq data of human lung adenocarcinoma tumors. **(c)** C2, C3 and C7 mRNA expression in metastases of human breast cancer from different metastatic sites. n=20, 22 and 18 for Lung, Brain and Bone groups, respectively. Data from GSE14020. P-values were calculated using One-way ANOVA with Tukey's correction for multiple comparisons. Source data are provided as a Source Data file.

Monteran_Ershaid_Supplementary Fig. 8

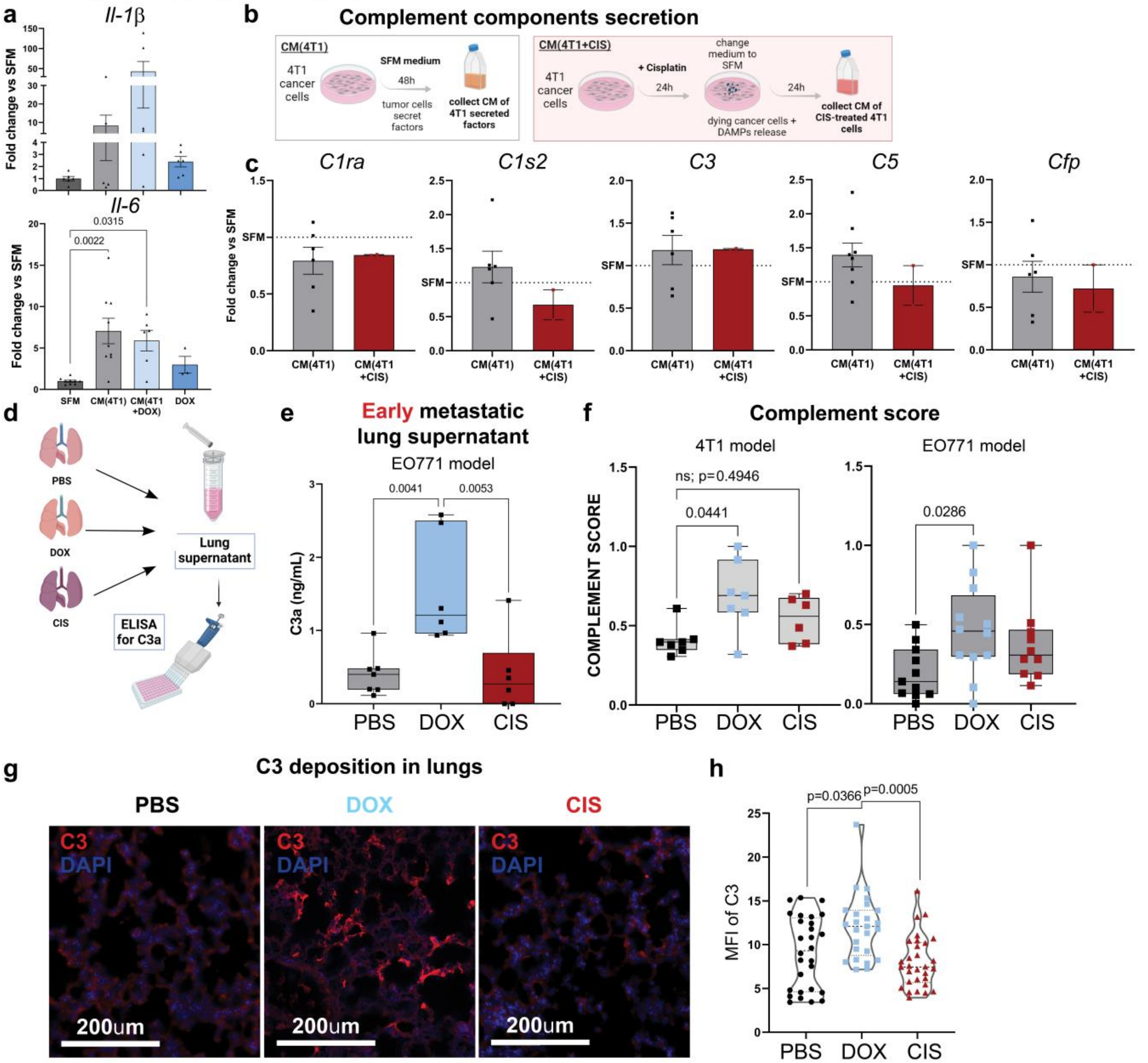

Supplementary Fig. 8. Complement-signaling activation occurs following doxorubicin, but not cisplatin treatment. (a) qRT-PCR analysis of inflammatory markers (*il6* and *il1b*) in primary lung fibroblasts. For *il1b* graph: n=6, 5, 6 and 6; and for *il6* graph: n=9, 9, 6 and 3 for SFM, CM(4T1), CM(4T1+DOX) and DOX alone, respectively. Data presented as mean ± s.d, normalized to SFM. P-values were calculated using One-way ANOVA. **(b)** Experimental scheme of indirect effects of cisplatin therapy on lung fibroblasts. Conditioned media was collected from cisplatin-treated 4T1 cells or from SFM-treated 4T1 cells. **(c)** qRT-PCR analysis of complement factors in lung fibroblasts treated as described in (b). n=6 and 2 for CM(4T1) and CM(4T1+CIS), respectively. Data are presented as mean ± s.d, normalized to SFM, and are representative of at least 2 biological repeats. P-values were calculated using One-way ANOVA. **(d, e)** C3a levels quantified by ELISA from early metastatic stage lungs in the EO177 model. n=7, 6 and 6 for PBS, DOX and CIS, respectively. P-values were calculated using one-way ANOVA. **(f)** Complement score of total lung mRNA from either 4T1- or EO771-injected mice at early metastatic stage. For the 4T1 model; n= 7, 7, and 6 for PBS,

DOX, and CIS respectively. For the EO177 model; n= 11, 12 and 10 for PBS, DOX, and CIS respectively. P-values were calculated using Kruskal-Wallis test. **(e, f)** Bars represent range, line represents mean. **(g)** Representative images of C3 staining in lungs of DOX, CIS or PBS treated mice at early metastatic stage. n=3 mice per group; Scale bars, 200µm. Cell nuclei-DAPI; C3-Rhodamine Red. **(h)** Quantification of MFI of C3 staining shown in (d), a minimum of 5 fields of view per lung were assessed. n=28, 25 and 30 for PBS, DOX and CIS, respectively. P-values were calculated using one-way ANOVA. In f-h, PBS and DOX data are shared with the experiment presented in main Fig. 6. Graphical summary was designed using BioRender. Source data are provided as a Source Data file.

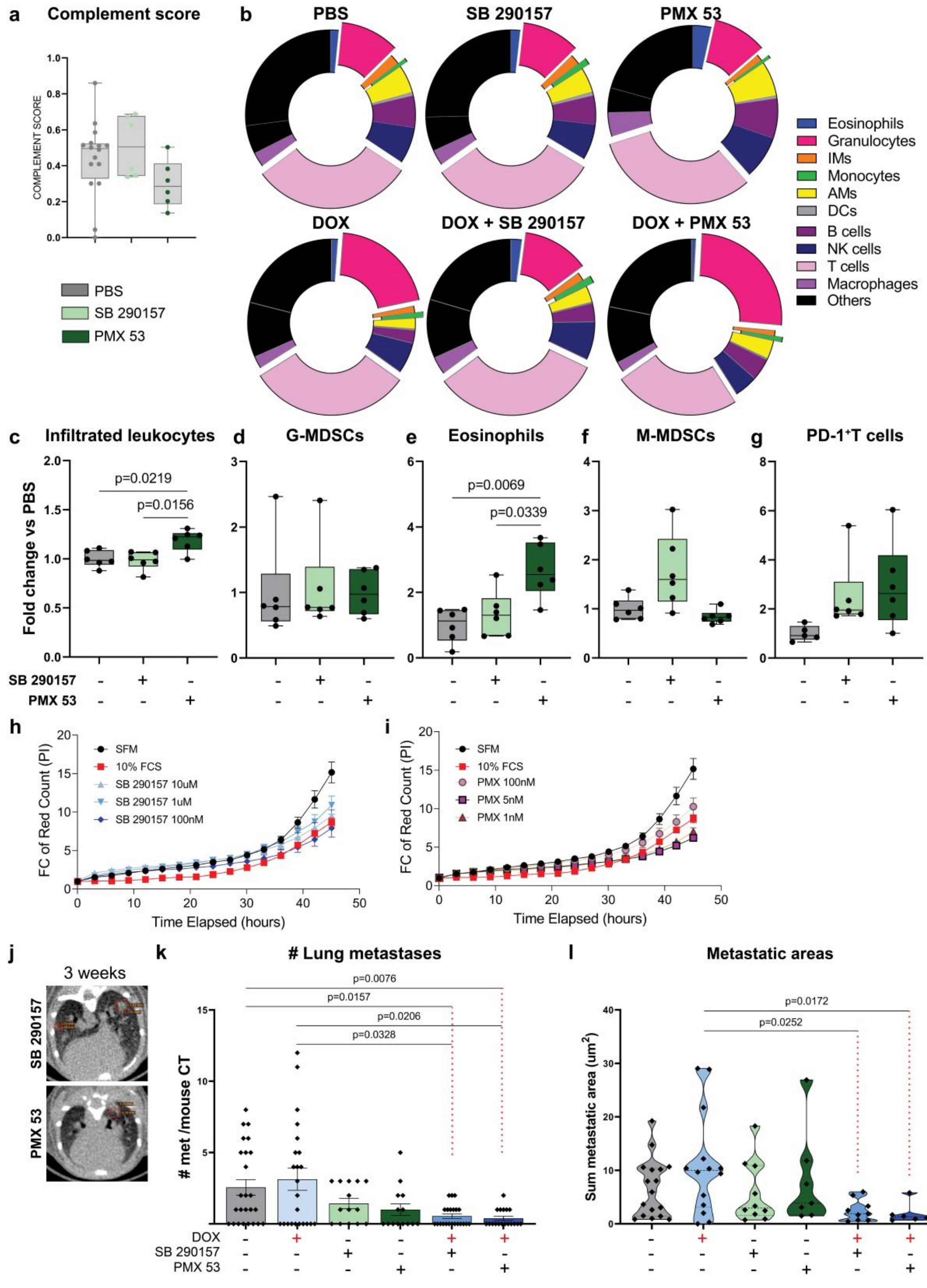

Supplementary Fig. 9. Complement blockade alone is inefficient in curbing pulmonary metastasis of breast cancer. (a) Complement score of total lung mRNA at early metastatic stage, 24h post treatment with SB290157 or PMX53, or PBS as control. n=16, 6 and 6 for PBS, SB290157 and PMX53, respectively. Bars represent range, line represents mean. P-values were calculated

using Brown-Forsythe ANOVA multiple comparison test. **(b-g)** Immune landscape of lungs at early metastatic stages following treatment with the specified regimen or PBS as control. **(b)** Donut plots depicting proportions of various immune cell populations. (**c-g**) Flow cytometry analysis of lung infiltrating immune cells at early metastatic stage. Bars represent range, line represents mean. n=6 mice per group. **(c)** Total immune cells ($CD45^+$ cells), **(d)** G-MDSCs, **(e)** Eosinophils, **(f)** Mono-MDSCs, **(g)** $PD1^+$ T cells. Data are presented as mean normalized to control. P-values were calculated using Kruskal-Wallis test for multiple comparisons. **(h, i)** Evaluation of cytotoxicity induced by complement antagonists. 4T1 cells were incubated with increasing doses of (h) SB290157 (100nM-10µM), or (i) PMX53 (1-100nM) for 48h. Serum-free media or medium with 10% FCS served as controls. Cytotoxicity was evaluated using PI staining measured by the Incucyte system. n=6 technical repeats, data are presented as mean ± s.e.m. P-values were calculated using One-way ANOVA test. **(j-l)** Quantification of lung metastatic burden. **(j)** Representative CT images depicting lungs 3 weeks after primary tumor. Metastatic lesions are circled in red**.** Quantification of lung metastatic foci n=25, 23, 14, 14, 22 and 18 for PBS, DOX, SB290157, PMX53, DOX+SB290157 and DOX+PMX53, respectively. **(k)** Metastatic area n=12, 8, 6, 5, 10 and 7 for PBS, DOX, SB290157, PMX53, DOX+SB290157 and DOX+PMX53, respectively. **(l)** Data are presented as mean ± s.e.m. P-values were calculated using Brown-Forsythe and Welch ANOVA tests for multiple comparisons**.** Data shown is from the experiments presented in Fig. 7 & 8. Source data are provided as a Source Data file.

Supplementary Table:

Supplementary Table. 1. Primer’s list.

Gene symbol	Forward sequence	Reverse sequence
Arg1	GGAATCTGCATGGGCAACCTGTGT	AGGGTCTACGTCTCGCAAGCCA
C1qa	GTGCCCGGCTTCTATTACTT	CCCGGAGGAAGACTTGATAAAC
C1qb	CCAGGATTCCATACACAGGAAG	AAACCTAGAAGCAGCAGTAACA
C1ra	AATCAAAGATGCAGAACGTGTATAG	GGTAAATGTTATTGCGGCTCTC
C1s1/C1s2	GCATACGACTCAGTGCAGATAA	CTGAGGTAAAGACCACCTGAAG
C2	ATGAGCTGGGTTCCAAGAAG	GTGAGCTTAGAGACATCCAACA
C3	GACCGAGCTAACCAACATAGAG	ATCTGAGCCTGACTTGATGAC
C3ar1	CCATCTCAGTGTGCTTGACTG	CATTGCCTAGCAGTCCCAATAG
C4a	GATGAGGTTCGCCTGCTATTA	CACATGGACACTCACGTTCTT
C4b	ACCCCCTAAATAACCTGG	CCTCATGTATCCTTTTTGGA
C5ar1	CTTCCTTCAGAAGAGTTGCCT	CTCCTGGGTTCTGTGGTAAC
C6	GCTTCCCACAAGAAGGATTCTA	TCTGAAAGCTGTAGGTCTGTTG
C7	GATGGATTTGTTCAAGGTGAAGG	CTGGCAACAGGATCTCCAATA
C8a	GTGGCATTGGCATAGGAATTAAG	CTTATGTCACGGTCACCATCTC
C8b	GAGGAGGGACAAGTGAGGATA	GGGCTCAGCCTTGATCTTAAT
C9	GAGACAGGCAGGTGCATAAA	GGTCATCGTCACAGTCATTCTC
Ccl3	GCTTTCTGCTCTTCAACACCAGATA	AGGAAAATGACACCTGGCTGG
Cd55	GCTTCATCCTGGTTGGAAATG	TGGGACCTTGGATTTCTCTATG
Cfb	CTCGGGCTCCATGAATATCTAC	TAACTCGCCACCTTCTCAATC
Cxcl2	CATCCAAAAGATACTGAACAAAGGC	TTTCTCTTTGGTTCTTCCGTTGA
CXCL5	GTTGTGTTTGCTTAACCGTAACT	GTTTAGCTATGACTTCCACCGTA
GAPDH	TGTGTCCGTCGTGGATCTGA	TTGCTGTTGAAGTCGCAGGAG
GUS	GCAGCCGCTACGGGAGTC	TTCATACCACACCCAGCCAAT
Hc = C5	GCTGACGCAGTCTGGATAAA	CACAGTTTGGCCTGGAGAATA
Hmgb1	GGACTCTCCTTTAACCGC	TTGTGATAGCCTTCGCTGGG
HPRT	TGATTATGGACAGGACTGAAAGA	GCAGGTCAGCAAAGAACTTATAG
Hspb1	GAAATACACGCTCCCTCCAG	GCCTCGAAAGTAACCGGAAT
Il1b	ACCCCAAAAGATGAAGGGCT	GATACTGCCTGCCTGAAGCTCT
Il6	ATACCACTCCCAACAGACCTGTCT	CAGAATTGCCATTGCACAACTC
LGALS9	AGTCTCCATACATTAACCCGATCA	TGGAGGGTCACCTGAAGTCC
Masp1	ACCTTCATGTCTGTCACTTTCC	CCTTGCACTCATCCACATCTAC
Masp2	CACCATGAGGCTACTCATCTTC	TACAGGTTCAGGCCACTTTG
Mbl2	GGGACAGAGCAGAATTTGATACTA	GAGAGAAGAGCACCCAGTTTC
MMP1a	AACTACATTTAGGGGAGAGGTGT	GCAGCGTCAAGTTTAACTGGAA
m-TNFa	CACCACGCTCTTCTGTCTACTG	GGAGGCCATTTGGGAACTTCT
Tgfb1	CTGAACCAAGGAGACGGAATAC	GGGCTGATCCCGTTGATTT
Tlr4	GTCCCTGATGACATTCCTTCT	TGTTTCAATTTCACACCTGGAT
UBC	GTTACCACCAAGAAGGTC	GGGAATGCAAGAACTTTATTC

vimentin	TTTCTTCCCTGAACCTGAGAGAA	GTCCATCTCTGGTCTCAACCGT
cxcl1	AGAATGGTCGCGAGGCTTG	CCGTTACTTGGGGACACCTTTTAG
NLRP3	CCCTTGGAGACACAGGACTCA	TGAGGCTGCAGTTGTCTAATTCC
TLR4	GTCCCTGATGACATTCCTTCT	TGTTTCAATTTCACACCTGGAT
Tlr3	CCCTCTGTGCAGAAGATTCAA	GCAAACAGAGTGCATGGTTTAG
ctgf	GTCAAGCTGCCTGGGAAATG	GTGTCTTCCAGTCGGTAGGCA
Col1α1	TGTGTTCCCTACTCAGCCGTCT	CTCGCTTCCGTACTCGAACG
TNC	GGACCTGCCTGGGCTCAA	CCAGCTGAGGGAGATCCTCTG
NOS2	TTCACCCAGTTGTGCATCGACCTA	TCCATGGTCACCTCCAACACAAGA
Arg1	GGAATCTGCATGGGCAACCTGTGT	AGGGTCTACGTCTCGCAAGCCA
Nox2	GACCCAGATGCAGGAAAGGAA	TCATGGTGCACAGCAAAGTGAT
PD1L1	AACACATCCTCCACAGAACAG	CTCCACATCTAGCATTCTCACTT
IL4ra	ACTGGATCTGGGAGCATCAA	GTAGTGTAGGCAGAGCTGAGAA
Fkbp5	GAAAGGCGAGGGATACTCAAA	CCACATCTCGGCAATCAAATG
Acta2	TCAGGGAGTAATGGTTGGAATG	GGTGATGATGCCGTGTTCTA
YM1	GCCACTGAGGTCTGGGATGC	TCCTTGAGCCACTGAGCCTTC
FIZZ1	CCTGCTGGGATGACTGCTA	TGGGTTCTCCACCTCTTCAT
VEGFA	CCACGACAGAAGGAGAGCAGAAGTCC	CGTTACAGCAGCCTGCACAGCG
il10	TGGCCCAGAAATCAAGGAGC	CAGCAGACTCAATACACACT
cfp	CTCCTGCTGCTACTGGTTATC	AGTCTTCTACCCTGATGTCTCT
RPS23	GATATTCCTGGAGTCCGCTTTA	ATGATCTTGGCCTTTCTTTCTTG
ACTB	CAGCCTTCCTTCTTGGGTATG	GGCATAGAGGTCTTTACGGATG
FLOT2	GGTGAAGATCATGACGGAGAAG	CTACAGTCAGAGTCCCTAGGATAG
mCherry	GAACGGCCACGAGTTCGAGA	CTTGGAGCCGTACATGAACTGAGG
